# Unveiling the psychosocial and academic implications of living with sickle cell disease among undergraduates in a private university in Nigeria

**DOI:** 10.3389/fpubh.2025.1531161

**Published:** 2025-01-31

**Authors:** Olumide T. Adeleke, Olufemi Folaranmi, Yetunde Olasinde, Larry Ayuba, Oludamola V. Adeleke, Modupe M. Ojo-Rufai, Efeturi Agelebe, Oyelola E. Adeoye, Aderemi T. Olabode, Dolapo E. Ajala, Titilola S. Akingbola

**Affiliations:** ^1^Department of Family Medicine, Bowen University Teaching Hospital/Bowen University, Iwo, Nigeria; ^2^Department of Haematology and Blood Transfusion, Bowen University Teaching Hospital/Bowen University, Iwo, Nigeria; ^3^Pediatric Department, Bowen University Teaching Hospital/Bowen University, Iwo, Nigeria; ^4^Nursing Unit, Bowen University Hospital, Bowen University, Iwo, Nigeria; ^5^Bowen University Hospital, Bowen University, Iwo, Nigeria; ^6^Public Health Programme, Bowen University, Iwo, Nigeria; ^7^Public Health Department, Fountain University, Osogbo, Nigeria; ^8^Haematology Department, University College Hospital/University of Ibadan, Ibadan, Nigeria

**Keywords:** sickle cell disease, academics, psychosocial, living with sickle cell disease, undergraduates, SCD

## Abstract

**Background:**

Sickle cell disease (SCD), a disease characterized with abnormal red blood cell morphology and is associated with diverse clinical manifestations and contribute to many psychosocial problems like social stigma, strained relationships and reduced self-esteem. SCD is highly prevalent in Sub-Saharan Africa, with Nigeria having the largest burden. This qualitative study is aimed at exploring possible psychosocial and academic challenges associated with sickle cell disease among undergraduates in Nigeria.

**Methods:**

From September 2023 to February 2024, the authors conducted an exploratory descriptive study using in-depth interviews among 22 undergraduate students with SCD at Bowen University, Osun State, Nigeria, employing an in-depth interviewer guide. Ethical approval was obtained from Bowen University Ethical Review Board prior to the conduct of the study. The data was analyzed using inductive thematic analysis.

**Results:**

Six (6) distinct themes emerged from the research data and each of the themes was linked to the study objectives. Many of the participants expressed disruption of academic activities by frequent SCD crisis. This negatively affected their academic performance, and sometimes led to feelings of frustration. Furthermore, the recurrent battles with pain due to vascular occlusion took a toll on their emotional and psychological health. They also experienced stigmatization and strained interpersonal relationships which negatively influenced their mental well-being. Conversely, some SCD patients enjoy some social support from colleagues and family members which gives hope and succor to them during difficult times.

**Conclusion:**

This study reveals that undergraduates with SCD face various academic and psychosocial challenges that affect their overall performance. The findings underscored the need for increased awareness, support, and understanding to better assist undergraduates with SCD in managing their health and academic responsibilities effectively. Considering the chronic nature of SCD and its myriads of psychosocial and academic challenges, measures should be put in place to mitigate the challenges and enable them to live a fulfilling life.

## Introduction

Sickle cell disease (SCD) is a group of haemoglobin disorders that have been acknowledged to have a global impact by the WHO (1, 2). It results from the synthesis of variant or mutant haemoglobins, resulting in abnormal red blood cell morphology (2). The sickle Haemoblogin (HbS) is formed from the substitution of valine for glutamic acid at position 6 of the *β*-globin chain of human hemoglobin (HbA). SCD results from homozygosity for this mutation or from a compound heterozygosity for HbS and *β*-thalassemia or another *β*-globin variant such as HbC, HbD, HbE, or HbO Arab (3, 4). Sickle cell disease is the most prevalent genetic disease in the African Region. It is estimated that over 300,000 babies are born with severe forms of hemoglobinopathies worldwide each year. While 75 percent of all patients with SCD live in Sub-Saharan Africa, Nigeria alone accounts for more than 100,000 new births every year (5). Nigeria has the highest burden of Sickle Cell anemia worldwide, and SCD is reported to pose an enormous social and financial burden on parents (6). It is characterized by chronic haemolysis, chronic anaemia, and recurrent painful crisis, with attendant psychosocial and academic challenges. Individuals with this disorder are also prone to strokes, recurrent infections, avascular necrosis, priapism, and growth delay (7).

Psychosocial challenges in patients living with SCD often result from recurrent painful crises and other symptoms affecting their daily activities. They may also experience some psychosocial challenges following medical complications of SCD such as leg ulcers, impaired growth, surgical scar, increased susceptibility to infection, and swollen hands and legs (8). These may inform society’s response towards people with SCD. However, some of these patients demonstrate emotional tolerance to the disease while others show feelings of frustration, anxiety, anger, resentment, and helplessness, occasioned by the prospect of being ill for the rest of their life (9). Other psychosocial challenges may include emotional upset and difficulty in getting a suitable partner in marriage (9). Patients with SCD have more school absences than others. This is largely due to recurrent crises, and frequent hospitalization. It has been reported that the academic performance of students with SCD is lower compared to their healthy counterparts, and this has been associated with diverse complications of the disease (10–14). King et al. also observed that Jamaican children with SCD had worse academic performance than their peers, with the male gender being more vulnerable (15). However, Alsalam et al. observed that patients with SCD are better educated than those without the disease (16). Alhazmi et al. also noted that students with SCD acknowledged better performance with online education during the COVID-19 era (14).

Previous studies have been largely quantitative, which do not explore the psychosocial and academic challenges of those with SCD in greater detail. The objective of this study, therefore, is to provide deep insight into their experiences which may aid our understanding of their various academic as well as psychosocial challenges and possible ways to assist them more in coping with such challenges.

## Methods

### Study design

This qualitative study employed an exploratory descriptive study design. We conducted in-depth interviews among undergraduate students studying at Bowen University, Osun State, Nigeria from September 2023 to February 2024. The choice of an exploratory descriptive design was made to allow for a comprehensive exploration of the research topic, capturing the nuanced experiences and perspectives of the participants. The qualitative exploratory in-depth interview with open-ended questions allowed for full exploration of the topic for contextual understanding.

### Study settings

The study was conducted at Bowen University, Osun State, Nigeria. Bowen University is a foremost private Christian university in Nigeria established by the Nigerian Baptist Convention in 2001. Participants were recruited purposively from their “Sickle Cells Support Group” in Iwo, Osun State, and Bowen University Teaching Hospital Haematology Clinic in Ogbomoso, Oyo State. This selection of settings was based on the nature of the research topic and the availability of participants, ensuring relevance and accessibility.

### Participants’ recruitment and sampling

Purposive sampling was employed to recruit participants from the targeted settings. Consenting students attending the Bowen University Sickle Cell Support Group during the study period were recruited. The sample size for the study was twenty-two (22) participants based on saturation. The undergraduates who consented were selected for an interview at an agreed time online. Saturation was reached at the twenty participants, however, two more participants were further interviewed to affirm the saturation.

### Data collection

Data collection involved in-depth interviews using a semi-structured guide to conduct virtual interviews using zoom platform to allow participants to express their views freely and enable researchers to seek clarifications using probes. The interviews lasted 40 min. The interviews were conducted online and recorded with permission from the participants. Field notes were also taken to record non-verbal clues. Recorded interviews were transcribed verbatim. The recorded audio files and documented field notes were reviewed for accuracy. Clarifications were sought from the participants where necessary. The study instrument was piloted at Ladoke Akintola University of Technology, Ogbomoso, Oyo State, Nigeria.

We fostered strong interpersonal connections, built trust, employed triangulation of data gathering by examining the phenomenon from different perspectives, sought feedback from participants to ensure interpretations reflected their perspectives, and performed a dependability audit.

### Reflexivity statement

We were able to guard our own biases, assumptions, opinions, beliefs, and presuppositions that might want to influence the study through reflexivity and bracketing. We ensured this by maintaining a reflexive journal during the research process, keeping notes and documentation of daily introspections which was beneficial and pertinent during the period of study. We presented the findings that emerged from the data as a true reflection of the perspectives of undergraduates with SCD about the academic and psychosocial implications of the disease.

### Data management

The data collected during the in-depth interviews were protected to maintain the confidentiality of the participants. Each interviewee was given a code based on the order of recruitment. Participants’ recorded and transcribed interviews were saved in the study folders. The recordings, transcript of the interviews, and field notes were kept in a safe locker separate from the demographic information and consent forms and made accessible to only the members of the researcher team. The stored raw data would be kept safe for five (5) years to guard against data loss.

### Data analysis

Analysis of the study data was done concurrently and in stages after each in-depth interview for accuracy and clarification from the participants. Data analysis adopted inductive thematic analysis, allowing for the identification, naming, and confirmation of emergent themes without preconceived thematic ideas. Transcripts were reviewed against audio files and field notes, coded, and analyzed for relationships and emerging patterns. Methodological rigour was ensured through sampling, piloting, and member checking, contributing to the credibility, transferability, and dependability of the study. Atlas-Ti software was used to aid the data analysis.

### Ethical considerations

Ethical clearance was sought and received from Bowen University Directorate of Research and Strategic Partnerships ethical review board with approval number: BUREC/COHES/FMD/004, ensuring the protection of participants’ rights and well-being. Participants were adequately informed of their right to withdraw consent for the study at any time. Confidentiality and anonymity were maintained through secure storage of the recorded tapes and field notes, with codes and pseudonyms utilized.

## Results

### Socio-demographic characteristics of the respondents

The result of this qualitative study is based on the twenty-two (22) in-depth interviews conducted among seven male and fifteen female undergraduate students in their university community. The age range is between 16 and 25 years. The study involved both male and female undergraduate students at various academic levels, ranging from 100 level to 400 level, final year students in other disciplines, and those in their clinical posting year at the College of Health Sciences in the university. Additionally, socio-demographic details included participants’ fields of study, such as medicine and surgery, anatomy, public health, environmental health science, architecture, mass communication, and law. Christianity and Islam are the two religious affiliations mentioned and the majority are from the Yoruba ethnic group.

### Summary of research results using thematic presentation

Six (6) distinct themes emerged from the research data and each of the themes was linked with the study objectives as presented below in the thematic summary table as shown in Table 1. The following sections explore some of the respondents’ comments in connection to the themes that were found.

**Table 1:** Table showing the objectives, thematic summary and representative quotes.

Objectives	Themes	Excerpts and quotes
Challenges imposed by SCD on participants’ academic performance	Disruption of individuals academic programme	“*It does affect my academic performance. Even the last time, I was at the clinic the Doctor asked me why I picked architecture. They did not tell you it is such a strenuous course? Sometimes, I have not slept all through the night…and then I have to rush for an 8 am class. And when I do come late, the lecturer just takes it as laziness…Generally speaking, yes, it has a very negative impact on my academics.” (Participant 14)*
	The need for special help and considerations from the university	*“There was a particular lecturer who used attendance. And because I was not feeling well, I was not always around in class. So by the time the results came out, I dropped from the first class that I already expected to a second class, which made me very unhappy.”(Participant 22)*
	Negative impact on overall academic performance	*“Like I even took a step back. My CGPA was on upper credit before I dropped to lower.” (Participant 10)*
Psychosocial challenges of SCD on participants	Negative impact on mental well-being	*“To be honest, I am not happy or proud to say I have this disease because mentally it affects me. I’m being drained mentally by this continuously because it’s not easy… And most times I’m like, God, did I ever offend you? …it’s just so painful.” (Participant 20)*
	Experience of mixed feelings	*“I would say that I also had self-esteem issues and I battled depression for a while for a very long period actually, even up until I think last year I had a relapse. But then I got better …I think that I just really went through every tough year of my life and I will say that God really helped me I’m much better than last year.” (Participant 22)*
	Stigmatisation and Interpersonal Relationship	*“The challenge may be the stigma that still comes with it, kind of the way people view you, the way they will look at you, that kind of thing. Those are part of the challenges…It used to make me feel down… If we look at the stigma, they are born out of ignorance.” (Participant 13)*

### Psychosocial implications of living with sickle cell disease

The participants’ expressions shed light on the multifaceted challenges faced by individuals living with sickle cell disease. These themes underscore the importance of addressing the psychosocial aspects of the illness, promoting mental health support, fostering understanding and empathy, and combating stigma to enhance the overall well-being and quality of life of individuals affected by sickle cell disease. The participants in this study shared compelling insights into the negative impact of sickle cell disease on their mental well-being, the experience of mixed feelings, and the challenges of stigmatization and strained interpersonal relationships.

#### Negative impact on mental well-being

Participants described the profound negative impact of sickle cell disease on their mental well-being. The constant battle with pain, fatigue, and the uncertainty of health crises took a toll on their emotional and psychological health. Feelings of anxiety, frustration, and helplessness were common as they navigated the challenges of managing a chronic illness while striving to meet academic and personal responsibilities. Some of their expressions were captured and are as follows:

*Sometimes the pain comes in a very excessive manner, that I would not be able to move and it stops me from doing what I want to do there are some times that I feel very down because the pain affects me also mentally. During that period, I would not want to do anything because I’d be feeling very down due to the excessive pain. But recently I’ve tried to motivate myself that even with the pain, it is just like a phase that shall soon pass. (Participant 1).*

*To be honest, I am not happy or proud to say I have this disease because mentally it affects me. I’m being drained mentally by this continuously because it’s not easy. The pain is not something I would wish someone to go through. And most times I’m like, God, did I ever offend you? …it’s just so painful. (Participant 20).*

*Most times, I feel angry and I’m just generally sad. I feel angry because I cannot do anything about it. It’s on rare occasions that it’s been cured. So seeing as this is the life I have been subjected to since I was about five years old, I shall continue to live like this till my time on this earth ends…(Participant 17).*

#### Experience of mixed feelings

The participants expressed experiencing a range of mixed feelings about their condition. While some shared feelings of gratitude for overcoming academic challenges and achieving success despite their health struggles, others recounted moments of despair, isolation, and emotional vulnerability. The juxtaposition of resilience and vulnerability underscored the complex emotional landscape of living with sickle cell disease.

*…my thoughts are not particularly friendly either. So I think I understand what you mean by the darker part of the emotion now. But this in any way led to any serious mental health problems. Okay, like seeing yourself being hopeless you know, maybe having low self-esteem or having to solve a mental health condition like depression. (Participant 10).*

*I would say that I also had self-esteem issues and I battled depression for a while for a very long period actually, even up until I think last year I had a relapse. But then I got better because I never really spoke to my parents because I did not know how they would take the news…I think that I just really went through every tough year of my life and I will say that God really helped me I’m much better than last year. (Participant 22).*

#### Stigmatization and interpersonal relationships

Participants highlighted the pervasive issue of stigmatization and its impact on their interpersonal relationships. The influence of religious-cultural beliefs and societal misconceptions led to feelings of shame, isolation, and judgment from others. Stigmatization not only affected how they viewed themselves but also strained their relationships with peers, friends, and even healthcare providers, creating barriers to seeking support and understanding.

*The challenge may be the stigma that still comes with it, kind of the way people view you, the way they will look at you, that kind of thing. Those are part of the challenges… Well, these days, I do not really get it again. But that time, of course, it used to make me feel down. That’s why these days, we have more people speaking out about sickle cell. So try to educate people on the right way to treat sickle cell patients, what to say, and what not to say. So this is getting better. If we look at the stigma, they are born out of ignorance. (Participant 13).*

*Well, for me, this is all a stigma. The way people might look down on you in society you might not be able to mingle with people that you choose to, you cannot be able to make friends and the rest of them so you will be withdrawn from conversations with your friends. (Participant 18).*

*The only people who are aware of my condition are my parents, my siblings, my immediate family, and then my caregivers here at school. So I do not get stigmatized, but I listen to other people who have conversations about sickle cell. And honestly, it is widely misunderstood. A girl in the school I attend has sickle cell disease as well. So my classmates were talking about it, and they were like, she’s an attention seeker. (Participant 14).*

Conversely, other participants conveyed their appreciation for the unwavering support and support they received from friends in the university residence halls and medical staff at the university hospital.

*“I want to thank God for my friends and roommates. They support me whenever I’m having a crisis with so much love, care, and prayers. The doctors particularly take care of me well. They manage my crisis well on campus” (Participant 11).*

### Sickle cell disease implications on participants’ academic performance

The participants’ narratives underscored the profound impact of sickle cell disease on their academic journeys, highlighting the significant disruptions to their academic programmes, the crucial need for special help and considerations from universities, and the detrimental effects on their overall academic performance. These themes emphasize the importance of creating inclusive and supportive academic environments that cater to the unique needs of students living with chronic health conditions like sickle cell disease. The participants in the transcript shared poignant accounts of how sickle cell disease significantly disrupted their academic programs, highlighting the need for special help and consideration from the university, and the negative impact on their overall academic performance.

#### Disruption of individuals academic programmes

Participants detailed how sickle cell disease disrupted their academic programmes, leading to missed classes, examinations, and academic activities. The unpredictable nature of the illness often resulted in sudden health crises that impeded their ability to consistently engage in their studies. These disruptions caused setbacks in their academic progress, creating challenges in keeping up with coursework and meeting academic requirements.

*It’s gonna really stress you, like, there was a time where I had an exam, and I had a crisis, okay, like, it’s very, very painful. But I had to just convince myself that I could do it. However, it lowered my marks because I was not able to focus on my studies. So I think this SS genotype probably affects your academic ability because there will be times when your body does not permit you to do things that are supposed to do when you are supposed to do (Participant 21).*

*Okay, so for me, it does have more negative effects. Okay. So, a couple of times, I’m struggling to pass because many times, I’ll miss classes because I’m having a crisis or anything……when I was not available, the first time I ever had a terrible crisis was when I had to do blood transfusions. By the time I came back to school, the lecturers already promised that they were going to do my makeup exam, I came back and they did different things. So they did not do a makeup exam. And then there was a particular person that used attendance and because I was not really feeling well, I was not always around in class. So by the time the results came out, I dropped from the first class that I already expected to a second class, which made me very unhappy (Participant 22).*

*It does affect my academic performance. Even the last time, I was at the clinic the Doctor asked me why I picked architecture. They did not tell you it is such a strenuous course? Sometimes, I have not slept all through the night, I’ll just record the general body pains, and then I have to rush for an 8 am class. And when I do come late, the lecturer just takes it as laziness. Or sometimes when setting deadlines, it’s us or the student’s negligence or, or sometimes you may even just make jokes about them like, Oh, if you like, do not take your academics seriously, maybe you think you depend on your parent’s money or something. Generally speaking, yes, it has a very negative impact on my academics (Participant 14).*

#### The need for special help and considerations from the university

The experiences of undergraduate students living with sickle cell disease underscore the importance of tailored support and considerations from the university community. Lecturers, colleagues, hostel managers, and the University Management all play a crucial role in providing special help and considerations that can have substantial impact on the academic journeys of these students. The narratives of students with sickle cell disease highlight the profound effects of the condition on their academic performance, emphasizing the need for inclusive and supportive academic environments that cater for their unique needs. To mitigate the detrimental effects of Sickle Cell Disease on academic outcomes, universities need to provide targeted support, flexibility, and understanding, ensuring that students with chronic health conditions like sickle cell disease can thrive and reach their full potential.

*There was a particular lecturer who used attendance. And because I was not feeling well, I was not always around in class. So by the time the results came out, I dropped from the first class that I already expected to a second class, which made me very unhappy (Participant 22).*

#### Negative impact on overall academic performance

The negative impact of sickle cell disease on the participants’ overall academic performance was evident in their accounts. Missed classes, exams, and the physical and emotional toll of managing the illness directly affected their ability to perform well academically. Participants shared experiences of academic setbacks, perceived neglect, and challenges in maintaining consistent academic progress due to the disruptions caused by the disease.

*There was a particular lecturer who used attendance. And because I was not really feeling well, I was not always around in class. So by the time the results came out, I dropped from the first class that I already expected to a second class, which made me very unhappy (Participant 22).*

*Like I even took a step back. My CGPA was on upper credit before I dropped to lower (Participant 10).*

## Discussion

This study offered powerful insights into the detrimental effects of sickle cell disease on the mental health of the study’s participants. It revealed the experience of mixed feelings and the difficulties associated with stigmatization and strained interpersonal relationships being experienced by people living with Sickle Cell Disease. The study also revealed that the profound impact of the illness on the participants’ academic performance was heavy, with missed classes and examinations due to health crises hindering their educational progress. These psychosocial effects and academic impact of sickle cell disease on the participants were evident, as they expressed feelings of frustration, and sadness, emphasizing the limitations in daily activities, serious disruptions to their specific academic programmes, the negative consequences on their overall academic performance, and the urgent need for university management to provide them with extra support and considerations. Several studies across the globe corroborated the findings that living with sickle cell disease has several psychosocial and academic implications (1, 14, 17–19).

The 22 participants involved in this study narrated their mental health ordeal with sickle cell disease, including anxiety, frustration, and helplessness, as they struggled to manage their chronic condition and meet academic and personal responsibilities. This negative impact of SCD on mental health is consistent with findings from other studies demonstrating that feelings of frustration, stress, and helplessness affect patients with SCD across the globe (20, 21). Therefore, it is imperative to address the great emotional burden experienced by many undergraduates with SCD. These negative mental health effects are typically caused by the ongoing struggle with pain, exhaustion, the unpredictability of health crises, and the difficulties in negotiating the disease’s psychosocial intricacies.

The study also revealed how the challenges of various perceptions of their condition by others have both negative and positive consequences on undergraduates living with Sickle Cell Disease. Negativity is more from the misconceptions and wrong perceptions of others about them and their condition resulting in stigmatization. This result aligns with earlier research findings that show the pervasive and deeply ingrained stigma associated with sickle cell disease (SCD), which can take many different forms (22, 23). Stigmatization presents physiological and psychological well-being challenges that have social consequences and may impact their overall quality of life. To create a more inclusive and supportive atmosphere for this susceptible group, it is, therefore, imperative to identify and address myths, misconceptions, and stigma surrounding SCD at personal, interpersonal, and institutional levels through focused education and awareness campaigns.

Ironically, the study also revealed the positive communal life in the hall of residence where others tend to be supportive of their perceived challenges and often give a helping hand. Importantly, most participants appear to have alluded to adequate and constant availability of support socially, medically, and religiously which comprehensively combined to serve as motivations for an enduring capacity to cope with the SCD conditions. This positive psychosocial effect confers the ability to endure and cope against the prevailing cultural nuances of stigma and the misconception of people living with SCD being labeled “Abiku”, “Ogbanje” (meaning licensed to die), attesting to a conquering of the religio-cultural factor that tends to be oppressive to those living with SCD.

Another significant finding that seems very crucial in this study has to do with the challenges of SCD participants missing lectures and in some cases examinations due to crises associated with the condition. Consequently, they often have lower grades when compared with their healthy counterparts as shown in a previous study by Alhazmi et al. Similar findings of school absenteeism and low academic performance of students with SCD have been documented in several studies (10, 24–26). However, this does not necessarily imply lower intelligence. When this happens, it would be wise for the administration of the university to allow them to retake the missed examinations at a later date. This will make it bearable and more conducive for such undergraduates living with SCD to cope. More supportive and structured educational services and policies are warranted to enhance the academic performance of this vulnerable population.

## Strengths and limitations

The study has several notable strengths that enhance its validity and reliability. To start with, the use of inductive thematic analysis allowed for a nuanced and detailed exploration of the research phenomenon, while reflexivity ensured that the researcher’s biases were identified and mitigated. The qualitative exploratory in-depth interviews with open-ended questions enabled rich and contextualized data to be collected, providing a deep understanding of the participants’ experiences and perspectives. The appropriate sample size achieved thematic saturation, and the use of Atlas-Ti software facilitated rigorous and systematic data analysis. Furthermore, the triangulation of data enhanced the study’s credibility and trustworthiness.

While the study has certain limitations, they are relatively minor and do not diminish from the overall quality of the research. The study’s focus on undergraduates at Bowen University, Iwo, Osun State, and Bowen University Teaching Hospital, Ogbomoso, Oyo State, may limit the generalizability of the findings to other populations. The virtual nature of the interview might limit the diversity of perspectives in the settings of poor internet access but the robust internet infrastructure in the University ensured that virtual interviews were conducted smoothly, mitigating potential disruptions. Additionally, while the study’s qualitative nature meant that quantitative data was not triangulated, the in-depth and contextualized data collected through the interviews provided a rich and detailed understanding of the research phenomenon, which is often not possible with quantitative methods alone. Nevertheless, the study’s findings provide valuable insights into the research topic, contributing to the existing body of knowledge and informing future research directions.

## Conclusion

This study concludes that undergraduates with SCD continue to face diverse academic and psychosocial challenges which invariably tend to impact their overall performances. However, there are some unstructured measures in place to mitigate the challenges experienced. Overall, the findings underscored the need for increased awareness, support, and accommodations to better assist undergraduate students with sickle cell disease in managing their health and academic responsibilities effectively.

### Recommendations

To expand on this study’s findings, future research should consider: conducting in-person interviews to boost the depth and nuance to the data, expanding the study to include a broader range of students to enhance generalizability, integrating quantitative data, such as psychosocial assessment scores and academic performance scores, to provide a more holistic understanding. Further effort should be targeted toward creating an enabling environment for undergraduates living with Sickle Cell Disease to optimize their academic performance and overall well-being.

## Data Availability

The raw data supporting the conclusions of this article will be made available by the authors, without undue reservation.

## References

[1] Anie KA Egunjobi FE Akinyanju OO. Psychosocial impact of sickle cell disorder: perspectives from a Nigerian setting. Glob Health. (2010) 6:2. doi: 10.1186/1744-8603-6-2, PMID: 20170540 PMC2836308

[2] Mburu J Odame I. Sickle cell disease: reducing the global disease burden. Int J Lab Hematol. (2019) 41:82–8. doi: 10.1111/ijlh.13023, PMID: 31069977

[3] Natrajan K Kutlar A. Disorders of Haemoglobin structure: sickle cell anaemia and related abnormalities In: Kaushansky K Lichtman MA Prchal JT Levi MM Press OW Burns LJ, editors. Williams Haematology: McGraw-Hill Global Education Holdings, LLC (1976). 1599–666.

[4] Ashutosh L Elliott PV. Sickle cell disease In: Hoffbrand VA Higgs DR Keeling DM Mehta AB, editors. Postgraduate Haematology. 7th ed. Oxford: Wiley Blackwell (2016). 98–113.

[5] Centre for Disease Control. Sickle Cell Disease. 2012. Available at: http://www.cdc.gov/globalhealth/countries/nigeria/what/scd.htm. accessed at22 /11 2017

[6] Olatunya OS Ogundare OE Fadare JO Oluwayemi IO Agaja TA Adeyefa BS. The financial burden of sickle cell disease on households in Ekiti. Southwest Nigeria Clinicoecon Outcomes Res. (2015) 7:545–53. doi: 10.2147/ceor.s86599 26622186 PMC4639479

[7] Wilkie DJ Johnson B Mack AK Labotka R Molokie RE. Sickle cell disease: an opportunity for palliative care across the life span. Nurs Clin North Am. (2010) 45:375–97. doi: 10.1016/j.cnur.2010.03.003, PMID: 20804884 PMC2932707

[8] Martinez RM Osei-Anto HA McCormick M National Academies of Sciences, Engineering, and Medicine. Complications of Sickle Cell Disease and Current Management Approaches In: Addressing Sickle Cell Disease: A Strategic Plan and Blueprint for Action. US: National Academies Press (2020) 33411430

[9] Adegboyega LO. Psycho-social problems of adolescents with sickle-cell anaemia in Ekiti state, Nigeria. Afric Health Sci. (2021) 21:775–81. doi: 10.4314/ahs.v21i2.37, PMID: 34795735 PMC8568230

[10] Al-Saqladi AW. The impact of sickle cell disease severity on school performance in affected Yemeni children. J Appl Hematol. (2016) 7:124. doi: 10.4103/1658-5127.198506

[11] Schwartz LA Radcliffe J Barakat LP. Associates of school absenteeism in adolescents with sickle cell disease. Pediatr Blood Cancer. (2009) 52:92–6. doi: 10.1002/pbc.21819, PMID: 19006248 PMC2684846

[12] Berkelhammer LD Williamson AL Sanford SD Dirksen CL Sharp WG Margulies AS. Neurocognitive sequelae of pediatric sickle cell disease: a review of the literature. Child Neuropsychol. (2007) 13:120–31. doi: 10.1080/09297040600800956, PMID: 17364569

[13] Jastaniah W. Epidemiology of sickle cell disease in Saudi Arabia. Ann Saudi Med. (2011) 31:289–93. doi: 10.4103/0256-4947.81540, PMID: 21623060 PMC3119971

[14] Alhazmi A Hakami K Abusageah F Jaawna E Khawaji M Alhazmi E. The impact of sickle cell disease on academic performance among affected students. Children. (2022) 9:15. doi: 10.3390/children9010015, PMID: 35053640 PMC8773889

[15] King LG Ali SB Chang SM Reid ME Soares DP. Academic performance in Jamaican children with sickle cell disease. J Natl Med Assoc. (2023) 115:475–81. doi: 10.1016/j.jnma.2023.07.005, PMID: 37550160

[16] Alsalman M AlHaddad S Alibrahim I Alabdullah AI Almutawa MH Alhamam AK. Impact of sickle cell disease on academic performance: a cross sectional study. eCollection. (2023) 17:2517–22. doi: 10.2147/PPA.S434750, PMID: 37841200 PMC10576454

[17] Heitzer AM Hamilton L Stafford C Gossett J Ouellette L Trpchevska A. Academic performance of children with sickle cell disease in the United States: a meta-analysis. Front Neurol. (2021) 12:786065. doi: 10.3389/fneur.2021.786065, PMID: 34966350 PMC8711768

[18] Harris KM Preiss L Varughese T Bauer A Calhoun CL Treadwell M. Examining mental health, education, employment, and pain in sickle cell disease. JAMA Netw Open. (2023) 6:14070. doi: 10.1001/jamanetworkopen.2023.14070, PMID: 37200033 PMC10196879

[19] Essien EA Winter-Eteng BF Onukogu CU Nkangha DD Daniel FM. Psychosocial challenges of persons with sickle cell anemia: a narrative review. Medicine. (2023) 102:e36147. doi: 10.1097/MD.0000000000036147, PMID: 38013366 PMC10681612

[20] Osunkwo I Andemariam B Minniti CP Inusa BP El Rassi F Francis-Gibson B. Impact of sickle cell disease on patientsʼ daily lives, symptoms reported, and disease management strategies: results from the international sickle cell world assessment survey (SWAY). Am J Hematol. (2021) 96:404–17. doi: 10.1002/ajh.26063, PMID: 33264445 PMC8248107

[21] Obeagu EI Asuma MN Tukur M. Counseling Services for Adolescents: nurturing mental health in sickle cell disease education. Elite J Public Health. (2024) 2:51–7.

[22] Bhat D Babu BV Surti SB Ranjit M Sarmah J Sridevi P. Stigma of sickle cell disease among Indian tribal population: a multi-centric qualitative study. J Natl Med Assoc. (2023) 115:556–65. doi: 10.1016/j.jnma.2023.09.006, PMID: 37845145

[23] Obeagu EI Obeagu GU. Addressing myths and stigmas: breaking barriers in adolescent sickle cell disease education. Elite J Health Sci. (2024) 2:7–15.

[24] Hassan M Abid FH Ahmed BAAH. School performance of children with sickle cell disease in Basra, Iraq. Iraqi J Hematol. (2019) 8:29. doi: 10.4103/ijh.ijh_21_18

[25] Olatunya OS Oke OJ Kuti BP Ajayi IA Olajuyin O Omotosho-Olagoke O. Factors influencing the academic performance of children with sickle cell Anaemia in Ekiti, South West. Nigeria J Trop Pediatr. (2017) 64:67–74. doi: 10.1093/tropej/fmx034 28549163

[26] Adeleke OT Olasinde YT Folaranmi OE Ayuba YL Agelebe E Rufai MM. A qualitative enquiry into lived experience and coping strategies of undergraduates with sickle cell disease in Nigeria. BMC Public Health. (2024) 24:1–8. doi: 10.1186/s12889-024-20927-6, PMID: 39639223 PMC11622687

